# Spinal disease diagnosis assistant based on MRI images using deep transfer learning methods

**DOI:** 10.3389/fpubh.2023.1044525

**Published:** 2023-02-24

**Authors:** Junbo Xuan, Baoyi Ke, Wenyu Ma, Yinghao Liang, Wei Hu

**Affiliations:** ^1^Guangxi Key Lab of Multi-Source Information Mining and Security, Guangxi Normal University, Guilin, China; ^2^School of Artificial Intelligence, Nanning College for Vocational Technology, Nanning, China; ^3^Department of Spine and Osteopathy Surgery, Guilin People's Hospital, Guilin, China

**Keywords:** spinal diseases, magnetic resonance image (MRI), image annotation, deep transfer learning, object detection, assisting doctor diagnosis

## Abstract

**Introduction:**

In light of the potential problems of missed diagnosis and misdiagnosis in the diagnosis of spinal diseases caused by experience differences and fatigue, this paper investigates the use of artificial intelligence technology for auxiliary diagnosis of spinal diseases.

**Methods:**

The LableImg tool was used to label the MRIs of 604 patients by clinically experienced doctors. Then, in order to select an appropriate object detection algorithm, deep transfer learning models of YOLOv3, YOLOv5, and PP-YOLOv2 were created and trained on the Baidu PaddlePaddle framework. The experimental results showed that the PP-YOLOv2 model achieved a 90.08% overall accuracy in the diagnosis of normal, IVD bulges and spondylolisthesis, which were 27.5 and 3.9% higher than YOLOv3 and YOLOv5, respectively. Finally, a visualization of the intelligent spine assistant diagnostic software based on the PP-YOLOv2 model was created and the software was made available to the doctors in the spine and osteopathic surgery at Guilin People's Hospital.

**Results and discussion:**

This software automatically provides auxiliary diagnoses in 14.5 s on a standard computer, is much faster than doctors in diagnosing human spines, which typically take 10 min, and its accuracy of 98% can be compared to that of experienced doctors in the comparison of various diagnostic methods. It significantly improves doctors' working efficiency, reduces the phenomenon of missed diagnoses and misdiagnoses, and demonstrates the efficacy of the developed intelligent spinal auxiliary diagnosis software.

## Introduction

The spine is the axial skeleton of the human body. Its role is to protect the spinal cord and bear weight. Its structure consists primarily of the vertebral body, intervertebral disc, vertebral arch, and posterior joints. It is also known as the “Second Lifeline” (1). Cervical spondylosis, spinal deformities, spinal degenerative disease, lumbar disc herniation, spinal tumors, and other spinal diseases are common and frequently occur in middle-aged and elderly patients (2). Currently, the diagnosis of spinal diseases is primarily based on clinical symptoms and medical images such as CT scans, MRI scans, and X-rays. It is unavoidable for doctors to combine personal subjective factors in the diagnosis process. It also takes a long time to label medical images for radiologists, and doctors are prone to missing a diagnosis or misdiagnosing patients due to fatigue. As a result, there is an urgent need for reliable automatic diagnosis technology for spinal diseases (3).

With the rapid advancement of computer technology and the advancement of medical imaging technology, medical image processing technology allows doctors to observe the pathological changes of the human body more directly and clearly, and can effectively assist or even replace the doctor in the relevant diagnosis. A growing number of researchers are using machine-learning techniques to diagnose spinal diseases (4, 5). Bounds et al. (6) used a multi-layer perceptron network trained to diagnose low back pain and sciatica with an accuracy of 77–82%, which was higher than the 68–76% of physicians. Ghosh et al. (7) used the intensity information and texture features of the disc in MRI images as classification conditions to build five classifiers that had the best support vector machine performance in diagnosing lumbar disc herniation. Castro-Mateos et al. (8) graded disc degeneration using an artificial neural network and a five-level grading system. However, in order to achieve higher accuracy, the classification algorithm must be optimized. The convolutional neural network (CNN) was used by Jamaludin et al. (9) and Shinde et al. (10) to grade the intervertebral disc and improve diagnostic efficiency. To calculate the Cobb angle of scoliosis, Zhang et al. (11) used a deep neural network to estimate the slope of the coronal spine. Wang et al. (12) used CNN to perform combined feature learning and independent feature learning on multi-view X-ray images, which they corrected before accurately estimating the Cobb angle of scoliosis. Wu (13) implemented the Pfirrmann classification and MSU classification diagnosis models and developed an automatic diagnosis system for lumbar disc protrusion based on deep learning technology, to provide patients and doctors with a basis for diagnosis and advice based on the degree of lumbar disc degeneration, position, and size of lumbar disc protrusion. As a result, the application of artificial intelligence technology for intelligent medical construction has emerged as a current research focus.

Deep transfer learning is the method of using deep learning for transfer learning and is a popular research direction of deep learning at present. Deep learning has two advantages over non-deep methods: first, it can extract more expressive features automatically, and second, it can meet the end-to-end requirements in practical applications. The auxiliary diagnosis of spinal diseases based on MRI scans belongs to the category of object detection. Object detection is a relatively mature model. In this way, this paper used the deep transfer learning method to assist doctors to diagnose spinal disease.

Guilin People's Hospital is a top-three hospital in China's northern Guangxi Zhuang Autonomous Region. The Department of Spine and Orthopedics is the hospital's most important department. With an increase in the number of patients with spinal diseases and a shortage of doctors with extensive clinical diagnosis experience, diagnosis can be inefficient, even leading to missed diagnoses or misdiagnoses. As a result, they intend to create intelligent spinal disease diagnosis software using artificial intelligence technology to assist doctors in improving diagnosis efficiency. So, the purpose of this paper is to investigate the application of deep learning technology in the diagnosis of spinal diseases based on doctors' practical work and to realize the cross-fusion and cooperative innovation of doctors and workers. It can help doctors improve the efficiency of diagnosis, improve the relationship between doctors and patients, optimize the service, and have better social benefits by designing and developing visual “End to end” intelligent spine disease diagnosis software.

The main work of this manuscript was to use artificial intelligence technology to develop and realize cross-collaborative innovation among doctors and workers. First, we collected the patients' MRI images for preprocessing, labeling of the image, and augmentation. Second, we selected the best model to develop the AI software by comparing the transfer learning YOLOv3, YOLOv5, and PP-YOLOv2 models. Finally, using the best-performing model, PP-YOLOv2, we developed the AI diagnosis software and provided a convenient “End-to-end” user interface for doctors.

## Materials and methods

### Spinal diseases image acquisition and desensitization

MRI images of 604 patients with spinal diseases were collected in routine clinical diagnosis from patients who visited the Department of Spine and Osteopathy Surgery of Guilin People's Hospital between July 2016 and July 2021, the main diseases included lumbar disc herniation (IVD bulges) and Spondylolisthesis. Each spinal MRI image had a resolution of 812 × 662 pixels. To protect the patient's privacy, the patient's MRI image data sets must be desensitized, which removes the patient's name, age, gender, and other privacy-related information. Patients' MRI image datasets were also collected for artificial intelligence training and auxiliary diagnosis only, and were approved by the Guilin People's Hospital Ethics Committee.

### Spinal MRI image labeling

**Figure 1:**
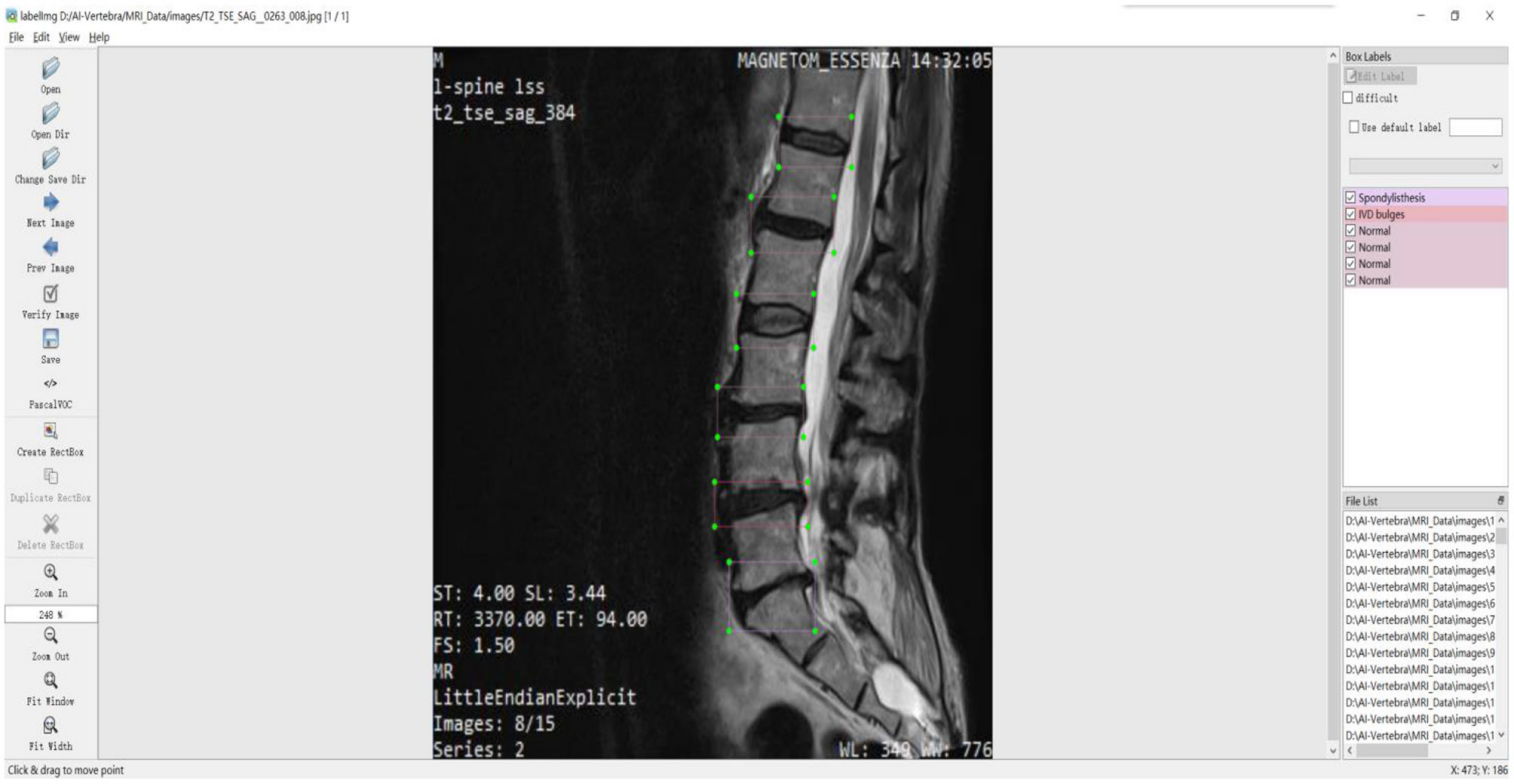
Schematic of spinal image annotation with the LabelImg software.

In deep learning, annotating data sets is a critical task that is related to the quality of training and the effect of prediction. The LabelImg tool is commonly used for image annotation. LabelImg is a visual image annotation tool that generates the data sets required by target detection algorithms such as Faster R-cnn (14), Yolo (15), and SD (16), and then generates the uniform format XML sample file for training. Image tagging of common spine diseases is required before MRI image datasets of patients with spine disease collected during the clinical process can be used for deep learning training. MRI image dataset disease type labeling was performed by spine surgeons with over 20 years of clinical diagnostic experience using the LableImg tool, ensuring high-quality image labeling training samples. The primary annotation types based on the type of disease collected from spine patients were normal, IVD bulges, and spondylolisthesis. These have different descriptions in clinical practice. IVD bulges are defined as a localized displacement of disc material beyond the margins of the intervertebral disc space. Spondylolisthesis is an acquired anterior displacement of one vertebra over the subjacent vertebra, associated with degenerative changes, without an associated disruption or defect in the vertebral ring. According to the definitions of normal, IVD bulges, and spondylolisthesis, the annotation of an MRI image schematic diagram is shown in Figure 1.

### Deep learning model analysis

Based on the actual situation of spine doctors' diagnosis work, the research team decided to select the target-based detection algorithm for development. In recent years, the YOLO Series (15) algorithm is a classical target detection algorithm.YOLO (You Only Look Once), as its name suggests, is a deep neural network-based object recognition and location algorithm. It runs faster and can be used in real-time systems. The YOLOv3 algorithm (17) and the YOLOv5 algorithm (18) are two representative algorithms in the YOLO series of target detection.

With the development of YOLO Algorithms, a new target detector named the PP-YOLO network model (19), based on the Baidu Deep Learning Framework PaddlePaddle was developed, which uses a series of optimization strategies based on YOLOv3, on the premise of hardly increasing the model parameters and computation. The precision of the detector is improved and the cost-effective single-stage target detector is obtained. The ResNet50vd-DCN model with deformable convolution, which has better precision and speed was one of the main optimization strategies. Furthermore, adding CoordConv and SPP and improving the efficiency of feature extraction through the methods of IOU Loss, IOU Aware, and so on, also optimizes the positioning accuracy of the detection frame based on the cross-union ratio (IOU).

One of the optimizations of the PP-YOLOv2 model (20) is to construct the detection neck of high-level semantic feature maps for various scale images. PP-YOLOv2 uses a path-based aggregation network to aggregate feature information from top to bottom. At the same time, by using the Mish activation function, the target area can be enlarged directly by increasing the input size, so that the network can capture the information of small-scale targets more easily and achieve higher performance. However, to reduce the memory size, the number of samples selected per training is doubled. In the case of the IOU Aware Branch, the target value is combined with the location confidence.

Only the IOU values of the positive samples are calculated.

Therefore, in order to select suitable deep learning models for development, the YOLOV3, YOLOv5, and PP-YOLOv2 models were applied to the MRI data sets of patients with spinal diseases to test and evaluate the true performance of each model.

### Deep transfer learning

**Figure 2:**
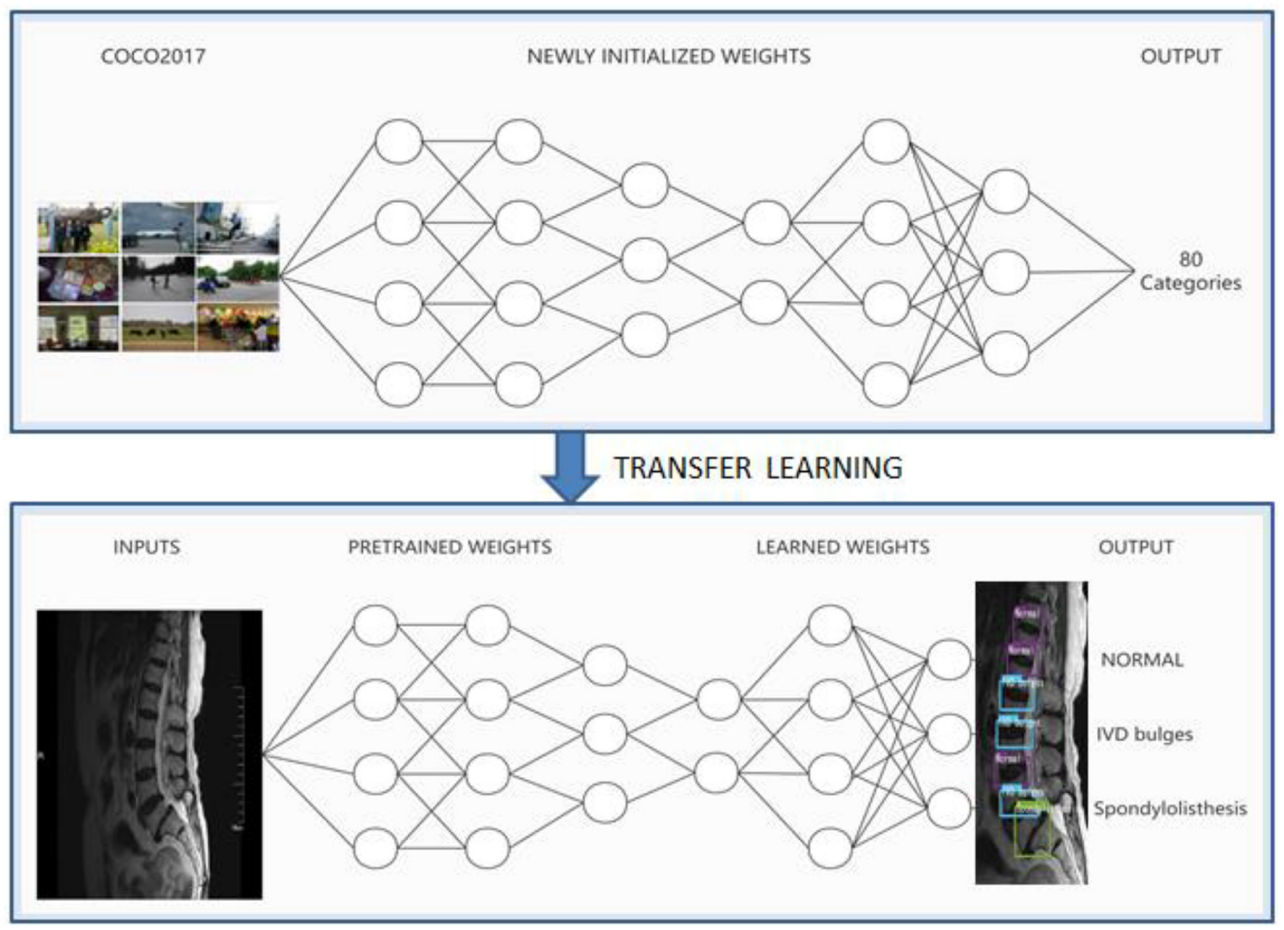
Schematic based on MRI and deep transfer learning.

Because the object detection models YOLOV3, YOLOv5, and PP-YOLOv2 were all trained on other common image data sets, the idea of transfer learning must be used to apply these models to the recognition and diagnosis of MRI images of patients with spinal diseases. The schematic based on MRI images and deep transfer learning is shown in Figure 2.

In the deep transfer learning method, the weight parameters obtained from the pre-training model are used to train the deep neural network on the MRI datasets labeled by doctors. When the deep neural networks learn about doctors' ability to diagnose disease, finally, the disease of each intervertebral disc is identified as normal, IVD bulges, or spondylolisthesis according to the label of the intervertebral disc.

Taking the PP-YOLOv2 model as an example, the algorithm process of the intelligent spine assistant doctor diagnosis research method based on the PP-YOLOv2 model is described as follows:

Step 1: the MS COCO2017 data set based on the model is used as the source domain detection task, and the pre-training is carried out, the parameters of the pre-training model were obtained;
Step 2: the target domain model was constructed as the source domain model;
Step 3: the MRI spine data set was used as the target domain detection task and the parameters of the pre-training model were used as the initial parameters of the target domain model to train the target domain model;
Step 4: the Adam algorithm was used to optimize the network;
Step 5: the detection and classification of the target were realized through the Logistic classifier.

**Figure 3:**
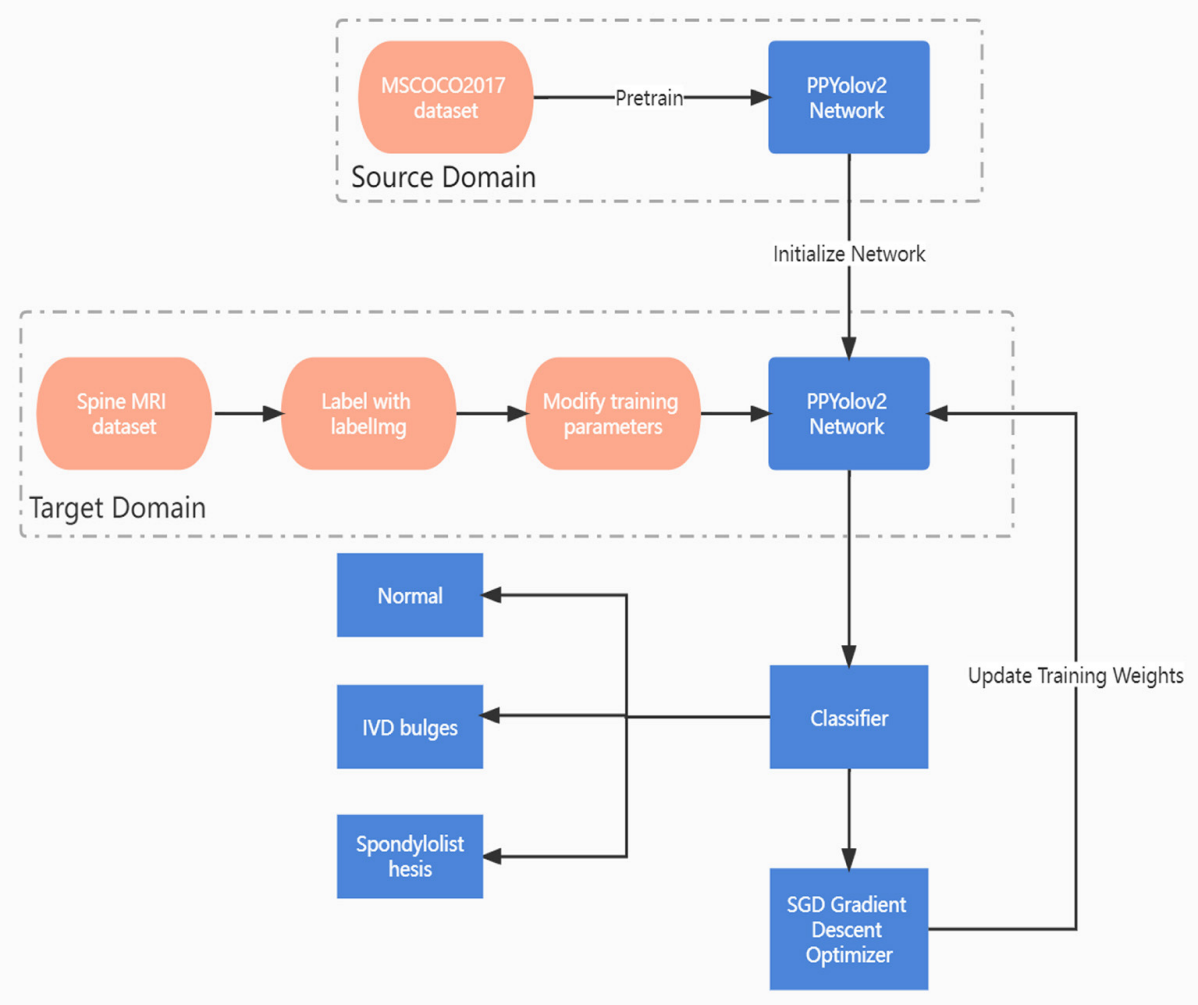
Flow chart of PP-YOLOv2 model based on MRI and deep transfer learning.

The flow chart of the PP-YOLOv2 model based on MRI images and deep transfer learning is shown in Figure 3.

## Results

### Experimental environment and parameters

The experimental environment for this article is Ubuntu 16.04, which is based on Baidu's deep learning framework PaddlePaddle2.0, and Python 3.6. The GPU is an Nvidia Tesla V100 with 32GB of memory.

Using a parameter adjustment strategy in the transfer learning process, a total of 1,800 epochs were set, using freeze training. In the first 300 epoch thawing backbone networks, only the detector layer was trained, with an initial learning rate of 0.00005, and then in the 301st epoch thawing training, the batch size of the input samples was 32 per iteration, and the gradient optimization function was performed using Adam. The attenuation coefficient is 0.0005, the momentum is 0.9, and the IOU threshold is set to 0.3.

### Data augmentation

Before the experiment of disease image classification, in order to ensure that different vertebral feature images have the same scale in the training set, verification set, and test set, it is necessary to perform image augmentation operations on the spine data set, including rotation angle, translation, flip and so on. The number of tags that were eventually labeled was 2,532 for normal, 1,467 for IVD bulges, and 585 for spondylolisthesis. To facilitate deep learning training, 80% of the spine data set used in the experiment was randomly selected as a training set,10% as a validation set, and 10% as a test set.

### Evaluation indicators

Precision, recall and mean average precision (mAP) were used to evaluate the effect of the deep learning model on the diagnosis of spinal diseases. The specific formula is defined as follows:


(1) $$\begin{array}{l} Precision=\frac{TP}{TP+FP} \end{array}$$

(2) $$\begin{array}{l} Recall=\frac{TP}{TP+FN} \end{array}$$

Among these, in the formula defined by the above accuracy and recall rates, TP (True Positives) indicates the number of spinal disorders diagnosed as positive and actually diagnosed correctly. FP (False Positives) denotes the number of spine disorders diagnosed as positive and actually misdiagnosed. FN (False Negatives) denotes the number of spine disorders diagnosed as negative and actually misdiagnosed.

In the process of spine disease diagnosis using the deep learning model, according to different confidence values, we can get different accuracy and recall. The P–R curve is drawn with the recall rate as the x-axis and precision as the y-axis, and the area covered under the curve is the average precision value of a diagnostic category. Assuming that m is the number of categories to be diagnosed, the mAP value of the model can be obtained by averaging the AP values of all the predicted diagnostic categories:


(3) $$\begin{array}{l} mAP=\frac{\overset{m}{\underset{i=1}{\sum }}AP_{i}}{m} \end{array}$$

### Experimental results and comparative analysis

In order to verify the validity and practicability of the PP-YOLOv2 model, the MRI data sets of spinal diseases were trained on three object detection models, which were YOLOv3, YOLOv5, and PP-YOLOv2. The mAP values of the three models were 70.64, 86.66, and 90.08%, respectively. The experimental results of different network models are shown in Table 1.

**Table 1:** The experimental result comparison of different models.

**Deep transfer learning model**	**Normal AP (%)**	**IVD bulges AP (%)**	**Spondylolisthesis AP (%)**	**mAP (%)**	**time(s)**
YOLOv3	81.17	69.06	61.68	70.64	16.2
YOLOv5	91.77	85.43	82.77	86.66	**13.7**
PP-YOLOv2	**93.84**	**91.74**	**84.67**	**90.08**	14.5

Bold values indicate the performance of every column evaluation indicator was the best.

**Figure 4:**
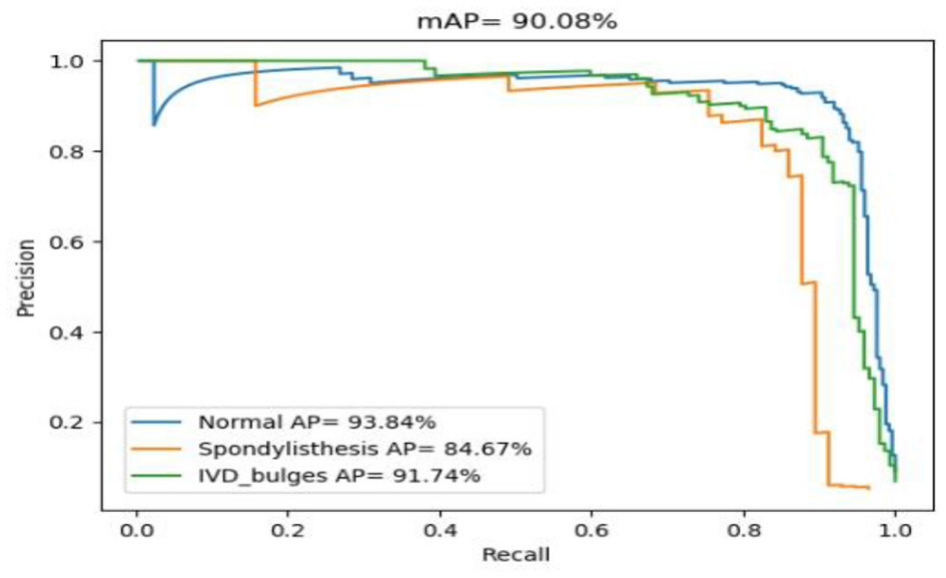
Diagnosis spine P–R curve based on the transfer learning PP-YOLOv2 model.

**Figure 5:**
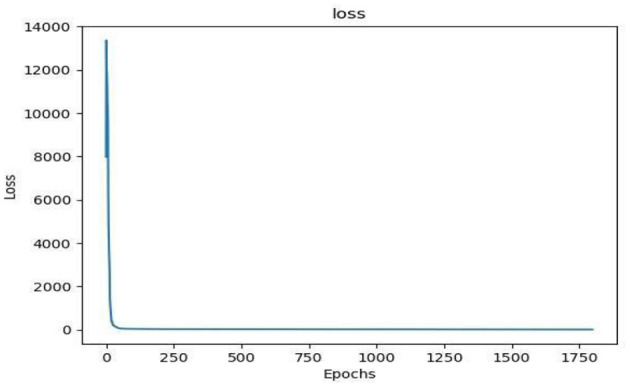
Loss function curve based on the transfer learning PP-YOLOv2 model.

As can be seen from Table 1, the PP-YOLOv2 model had the highest accuracy in diagnosing normal, IVD bulges, and spondylolisthesis compared with the YOLOv3 and YOLOv5 models, at 93.84, 91.74, and 84.67% respectively. At the same time, the overall accuracy of mAP was 90.03%, which was also the highest, 3.9 and 27.5% higher than that of YOLOv5 and YOLOv3, respectively. An MRI image of a spinal patient takes about 14.5 s to diagnose on a common computer, much faster than the average 10 min that a spinal surgeon would need to manually diagnose. The P–R curve and the loss function curve of the PP-YOLOv2 model are shown in Figures 4, 5 respectively.

In Figure 4, the area under the P–R curve for each spinal category is the accuracy of that category. The blue P–R curve indicates that the diagnostic accuracy of normal was 93.84%, and the cyan P–R curve indicates that the diagnostic accuracy of IVD bulges was 91.74%. The orange P–R curve indicates an accuracy rate of 84.67% for the diagnosis of spondylolisthesis, which is due to the fact that patients with spondylolisthesis are less prevalent than normal and lumbar disc herniation in daily diagnosis. There were also fewer labeled data sets in the spondylolisthesis collection, so the accuracy was lower than in the other two categories.

As can be seen in Figure 5, the PP-YOLOv2 model converges quickly during training, indicating that the diagnostic model obtained from the training dataset with this model is better, and therefore, the trained PP-YOLOv2 model was used to develop a visual platform for spine auxiliary diagnosis.

### Development of visual assistant diagnosis software

As previously shown, the PP-YOLOv2 model greatly improves the accuracy of spine disease diagnosis by optimizing the network structure as well as unique algorithms. It can assist doctors to make rapid and accurate diagnoses of spinal diseases. Therefore, in order to assist doctors to diagnose spinal diseases more conveniently, a visual intelligent spinal assistant diagnosis software was developed using the spinal disease prediction model trained by the PP-YOLOv2 algorithm. In the concrete realization of the software the PyQt framework, Flask Web framework, and Android WebView technology are used to implement the PC, Web, and mobile application interfaces of the intelligent spine assistant diagnosis software, respectively. The software interface is simple and friendly.

**Figure 6:**
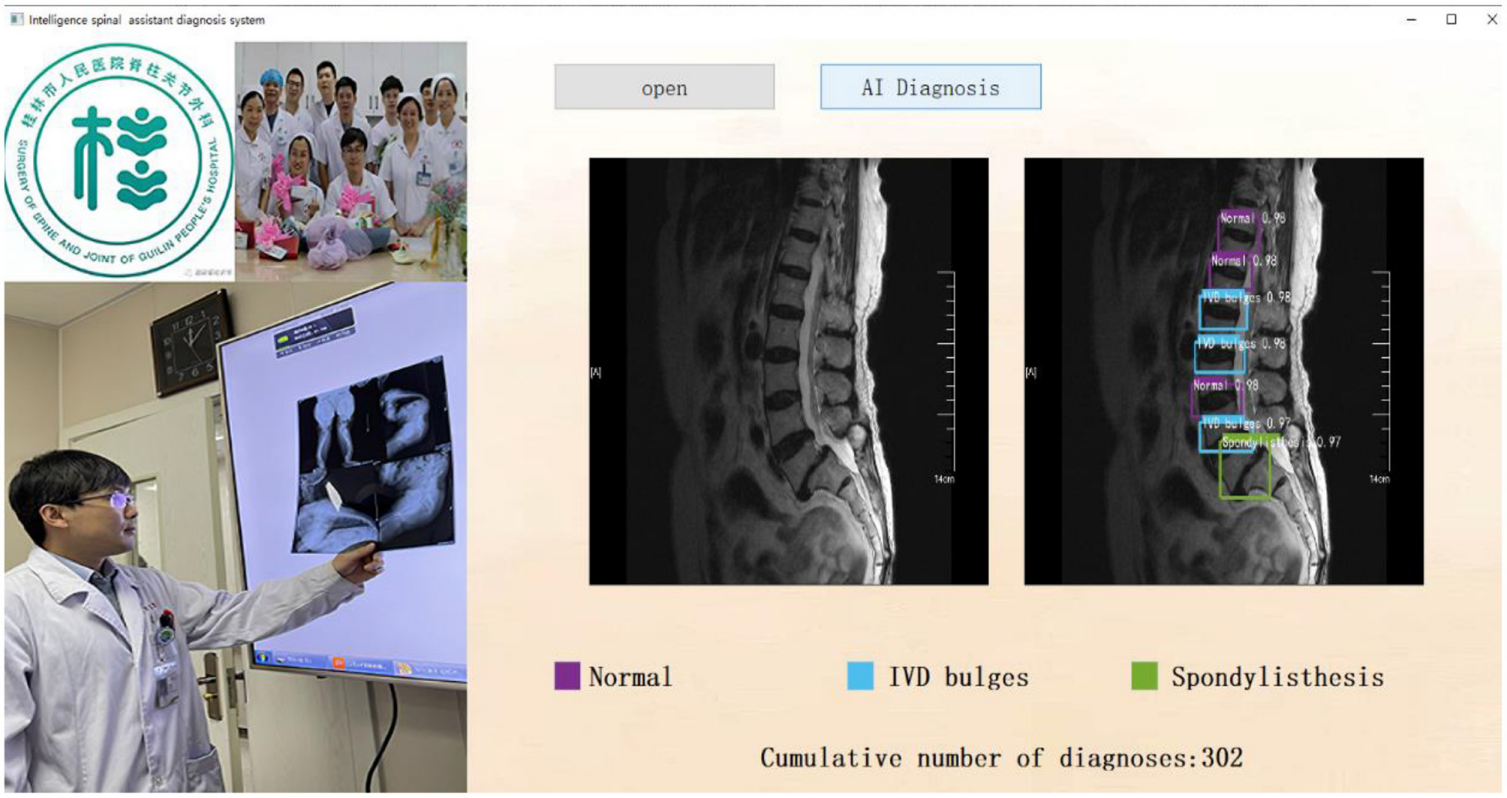
Intelligence spinal assistant diagnosis system (PC version).

By constructing the auxiliary diagnosis software, we provide an “End-to-end” visual auxiliary diagnosis interface for spine surgeons, and only need to obtain one MRI image of patients with spinal diseases. An auxiliary diagnosis result can be given automatically after ~14.5 s on a common computer. The design and implementation of the PC version of the auxiliary doctor diagnosis system are shown in Figure 6.

### Comparison of AI systems and doctors with different years of experience

To facilitate comparison, three doctors with varying levels of seniority were chosen from the Department of Spine and Orthopedics at Guilin People's Hospital. They were designated as Physician 1 (rotated for 6 months in the spinal department), Physician 2 (postgraduate with 2 years of experience in the spinal department), and Physician 3 (with 8 years of experience in spinal disease diagnosis). The intelligent spine diagnosis system was designated as the AI system.

When specific experimental test comparisons were made, 50 patients with spinal diseases were first selected for MRI images include 123 IVD bulges, 70 spondylolisthesis, and 198 normal spines, which were confirmed by 3 experienced spine surgeons. The AI System and physicians 1, 2, and 3, respectively, diagnosed the 50 cases of patient images and recorded the diagnosis. The IBM SPSS 23.0 software was used to analyze the sensitivity, specificity, and accuracy of AI systems and the physicians of different experience levels in the diagnosis of normal, IVD bulges, and spondylolisthesis. All data were tested using the chi-square test, with *P* < 0.05 as the statistically significant difference. The results of the comparative analysis of the experimental tests are shown in Tables 2, 3.

**Table 2:** Diagnostic efficacy in the diagnosis of normal and diseased disks.

**Spinal doctor**	**Sensitivity**	**Specificity**	**Accuracy**
Physician1	74.75% (148/198)	93.78% (181/193)	84.14% (329/391)
Physician2	76.77% (152/198)	92.75% (179/193)	84.65% (331/391)
Physician3	90.91% (180/198)	94.30% (182/193)	92.58% (362/391)
**AI system**	**97.98% (194/198)**	**98.45% (190/193)**	**98.21% (384/391)**

Bold values indicate the performance of every column evaluation indicator was the best.

**Table 3:** Diagnostic efficacy in the diagnosis of normal and IVD bulges.

**Spinal doctor**	**Sensitivity**	**Specificity**	**Accuracy**
Physician1	74.75% (148/198)	63.41% (78/123)	70.40% (226/321)
Physician2	76.77% (152/198)	79.67% (98/123)	77.88% (250/321)
Physician3	90.91% (180/198)	90.91% (108/123)	87.80% (288/321)
**AI system**	**97.98% (194/198)**	**98.37% (121/123)**	**98.13% (315/321)**

Bold values indicate the performance of every column evaluation indicator was the best.

From the data in Table 2, the specificity of the AI system was higher than that of the three doctors in the diagnosis of normal and abnormal intervertebral disc (*P* = 0.061), and there was no significant difference between the AI system and the three doctors in the diagnosis of normal and abnormal intervertebral disc (*P* = 0.061). The sensitivity, specificity, and accuracy of the AI diagnosis were 97.98, 98.45, and 98.21%, respectively, and that of the physicians were 90.91, 94.30, and 92.58%, respectively. The diagnostic sensitivity and accuracy of physician 1 and physician 2 were lower (*P* < 0.05), and the diagnostic specificity and accuracy of the AI system were significantly different from those of physician 1, physician 2, and physician 3 (*P* < 0.05).

According to the data in Table 3, the sensitivity, specificity, and accuracy of the AI system in the diagnosis of normal and IVD bulges were significantly different from those of the three physicians (*P* = 0.000). The diagnostic efficiency of the AI system was the best because its diagnostic sensitivity (97.98%), specificity (98.37%), and accuracy (98.13%) are the highest, physician 3 was second, and physicians 2 and 1 were the lowest.

As a result of the comparison between Tables 2, 3, the AI system achieves superior diagnostic performance in the auxiliary diagnosis of spinal diseases.

### Application of spine assistant diagnosis software

**Figure 7:**
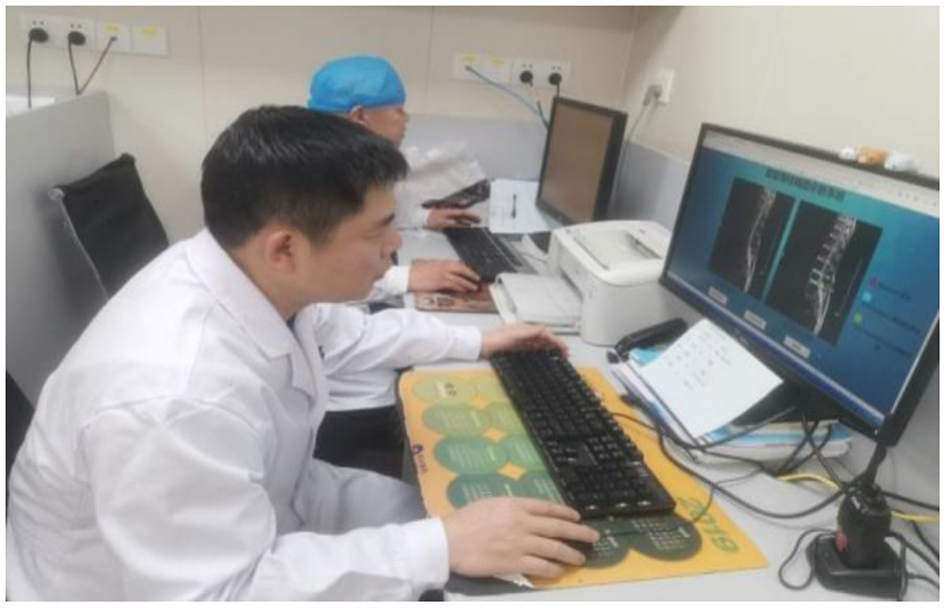
Guilin People's Hospital doctors use the intelligence spinal assistant diagnosis software.

After extensive testing and feedback from spine surgeons, the intelligent spine-assist doctor diagnostic software developed by the PP-YOLOv2 deep learning model can match the diagnostic accuracy of experienced senior spine surgeons while performing significantly better than junior spine surgeons. On January 10, 2022, the diagnostic software was deployed in the Guilin People's Hospital and was being used to assist orthopedic surgeons in their daily clinical diagnosis. Doctors use software to aid diagnosis as shown in Figure 7.

## Discussion

This research collected patients' MRI images to only include the three categories of normal, IVD bulges, and spondylolisthesis. Then, AI technology was used to learn the doctors' diagnoses for the three categories. This research used artificial intelligence technology to develop and realize cross-collaborative innovation among doctors and workers and provided a convenient “End-to-end” user interface for doctors. The results of the practical application in the hospital show that the intelligent spinal assistant diagnosis software developed with artificial intelligence technology has greatly improved the work efficiency of doctors. It can reduce missed diagnoses and misdiagnoses caused by a shortage of doctors, experience difference or fatigue, improve the relationship between doctors and patients, optimize the service for patients with spinal diseases, and produce remuneration. The software can also be used conveniently in grass-roots hospitals to provide high-quality medical resources. However, this research does not collect enough patient data, and the types of diseases diagnosed are limited.

## Conclusion

This paper uses artificial intelligence technology to develop and realize cross-collaborative innovation among doctors and workers, which meets the needs of current intelligent medical construction, based on the daily diagnosis needs of orthopedics doctors at the Guilin People's Hospital. MRI image data sets of spine patients were collected by doctors, labeled after desensitization, and then an artificial intelligence professional team designed and developed a visual intelligent spine auxiliary diagnosis software using deep learning technology, providing a convenient “End-to-end” user interface for doctors. The software's practical application demonstrates that the software can provide an auxiliary diagnosis result in ~14.5 s for an MRI image of a patient with spinal disease, and the accuracy rate is 90.08%, which can be compared to expert doctors, and greatly improve the efficiency of diagnosis, reduce the risk of missed diagnosis or misdiagnosis, and provide better social benefits. However, this advancement does not collect enough patient data, and the types of diseases diagnosed are limited. The next step is to continue collecting patient data sets for training in order to improve the accuracy and expand the disease types covered by physician-assisted diagnosis.

## Data availability statement

The original contributions presented in the study are included in the article/supplementary material, further inquiries can be directed to the corresponding author.

## Ethics statement

The studies involving human participants were reviewed and approved by the Ethics Committee of Guilin People's Hospital. The patients/participants provided their written informed consent to participate in this study. Written informed consent was obtained from the individual(s) for the publication of any potentially identifiable images or data included in this article.

## Author contributions

JX and BK contributed to the conception and design of the study and wrote sections of the manuscript. WM organized the database and annotation. YL coded and developed the AI System. BK and WM performed the statistical analysis. JX wrote the first draft of the manuscript. JX and WH revised the manuscript. WH supervised the study. All authors contributed to the manuscript revision, read, and approved the submitted version.
